# Supplementary material for: Heterobimetallic Ru(II)/M (M = Ag+, Cu2+, Pb2+) Complexes as Photosensitizers for Room-Temperature Gas Sensing
Source: Molecules. 2022 Aug 9;27(16):5058. doi: 10.3390/molecules27165058 (PMC9415935; doi:10.3390/molecules27165058)
Supplement: Supplementary file 1 [file molecules-27-05058-s001.zip › molecules-1854062-supplementary.pdf]

Supplementary Materials

Heterobimetallic Ru(II)/M (M = Ag^{+}, Cu^{2+}, Pb^{2+}) Complexes as Photosensitizers for Room-Temperature Gas Sensing

Abulkosim Nasriddinov [1,2], Sergey Tokarev [3], Vadim Platonov [1], Anatoly Botezzatu [3], Olga Fedorova [3], Marina Rumyantseva [1] and Yuri Fedorov [3,*]

1 Chemistry Department, Moscow State University, Moscow 119991, Russia
2 Faculty of Materials Science, Moscow State University, Moscow 119991, Russia
3 A.N. Nesmeyanov Institute of Organoelement Compounds RAS, Moscow 119991, Russia
* Correspondence: fedorov@ineos.ac.ru; Tel.: +7-(499)-135-92-80

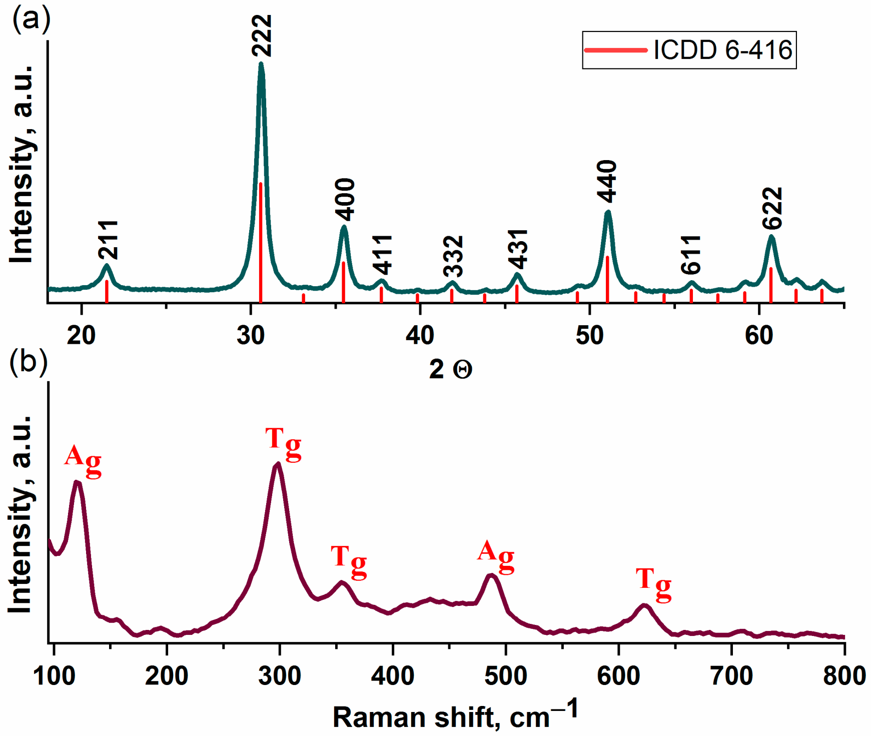

Figure S1. XRD pattern (a) and Raman spectrum (b) of nanocrystalline In_2O_3.

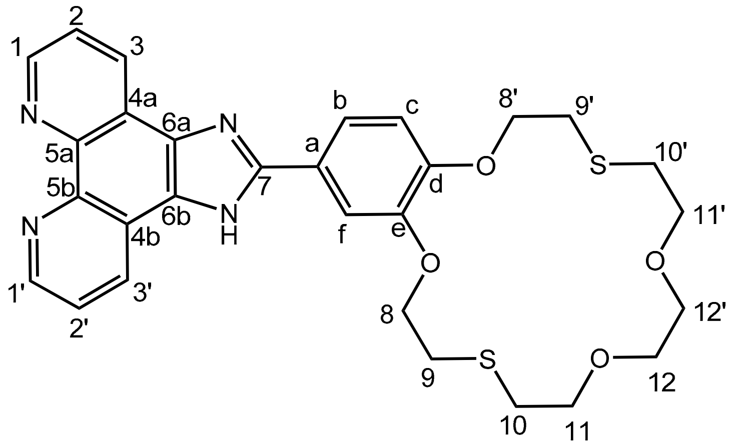

Figure S2. Structure of the *2-(2,3,5,6,8,9,11,12,14,15,16a,20adodecahydrobenzo[b] [1,4,10,13,7,16] tetraoxadithiacyclooctadecin-18-yl)-1H-imidazo[4,5- f][1,10]phenanthroline*. (**2**)

1,10-phenanthroline-5,6-dione (0.9 mmol, 191 mg), ammonium acetate (18 mmol, 1.4 g), and 2,3,5,6,8,9,11,12,14,15,16a,20a- dodecahydrobenzo[*b*][1,4,10,13,7,16]tetraoxadithiacyclooctadecine-18-carbaldehyde (0.9 mmol, 372 mg), were dissolved in 25 mL of glacial acetic acid. The mixture was stirred for 20 hours at reflux. An aqueous solution of NH_4OH (13%) was added to pH 8. The precipitate formed was filtered on a glass porous filter and washed with water and diethyl ether. After washing, the sediment was boiled in 10 mL of methyl alcohol for 30 minutes, filtered and dried on a rotary evaporator at 75 ^{o}C 1h. The pure product was obtained as a beige precipitate (360 mg, 0.64 mmol). Yield 71%. ^{1}H NMR (DMSO – d6, δ; m.d., J/Hz): 2.89 (m, 4H) H(10,10′); 3.04 (t, 2H, ^{3}J=6.4) H(9′); 3.09 (t, 2H, ^{3}J=6.5) H(9); 3.49 (s, 4H) H(12,12′); 3.61(m, 4H) H(11,11′); 4.2 (m, 4H) H(8,8′), 7,13 (d, 1H, ^{3}J=8.5) H(c), 7.78 (m, 4H) H(b,f,2,2′), 8.86 (m, 2H) (H(3,3′); 8,98(m, 2H) (H1,1′) 13.47 (s, 1H) (NH). ^{13}C NMR (DMSO-d6, δ; m.d., J/Hz): 30.59, 30.64 (2C) (9,9′); 31.24, 31.29 (2C) (10,10′), 69.93, 69.95, 70.02 (4C) (8,8′,12,12′), 72.01, 72.21 (2C) (11,11′), 110,43 (1C) (f), 112.82 (1C) (c), 119.70, 119,77 (2C) (a,b), 123.14, 123.47, 123.77, 124.14 (4C) (2,2′, 5a,5b), 130.03 (2C) (3,3′), 143.75, 143.99 (2C) (4a,4b), 148.02, 148.11 (2C) (6a, 6b),148.08 (2C), (1,1′) 148.35 (1C) (e), 149.56 (1C) (d), 151.16 (1C) (7). ESI-MS m/z (Iотн, (%)): 563.2 [**2**+H]$^{+}$. Calculated for $C_{29}H_{30}N_4O_4S_2$ (%): C, 61.90; H, 5.37; N, 9.96; found (%): C, 62.07; H, 5.22; N, 10.09;

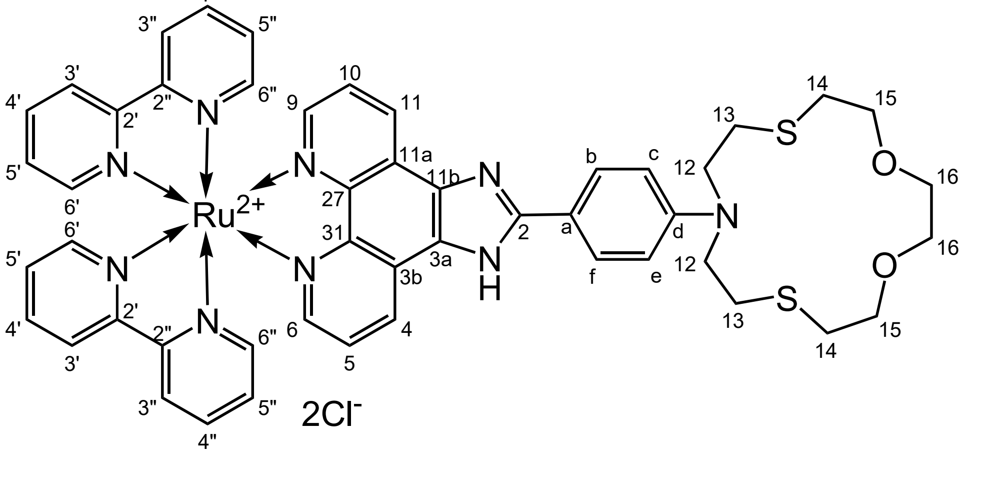

Figure S3. Structure of the *bis(2,2-bipyridine-k⁴N¹′,N¹″)[2-(10-(4-(1H-imidazo[4,5-f][1,10]phenanthrolin-2-yl)cyclohexa-2,4-dien-1-yl)-1,4-dioxa-7,13-dithia-10-azacyclopentadecane)-1H-imidazo[4,5-f][1,10]phenanthroline-k²N⁷,N⁸]ruthenium(II) chloride.* (**3**)

cis-bis(2,2- bipyridine)dichlororuthenium (II) hydrate (0.0915 mmol, 43.6 mg) and ligand **1** (0.0915 mmol, 50 mg) were dissolved in 3 ml absolute ethanol. The solution was placed in a tube, degassed 3 times and filled with argon. The tube was sealed and stirred at 80°C for 8 hours. Next, the tube was opened and the solvent was evaporated on a rotary evaporator. The resulting crude product was purified by flash column chromatography on alumina, eluent – a mixture of benzene:methanol in a ratio of 5:1 to obtained 58 mg (0.0563 mmol) scarlet precipitate. Yield 62 %.^{1}H NMR (CD_3OD - d4, δ; m.d., J/Hz): 2.80 (t, 4H, ^{3}J=5.2) (14); 2.94 (t, 4H, ^{3}J=7.4) (13); 3.68 (s. 4H) (16); 3.76 (t, 4H, ^{3}J=7.5) (12); 3.82 (t, 4H, ^{3}J=5.2) (15); 6.78 (d, 2H, ^{3}J=8.4) (c,e), 7.32 (m, 2H) (5′); 7.56 (t, 2H, ^{3}J=6.7) (5′′); 7.71 (d, 2H, ^{3}J=5.4) (6′); 7.86 (dd, 2H, ^{3}J=3.3, ^{3}J=5.2) (5,10); 7.96 (d, 2H, ^{3}J=5.4) (6′′), 8.06 (m, 2H) (b,f), 8.09 (m, 2H) (4,11); 8.09 (m, 2H) (4′); 8.19 (t, 2H, ^{3}J=7.8) (4′′); 8.71 (d, 2H, ^{3}J=8.3) (3′); 8.77 (d, 2H, ^{3}J=8.3) (3′′); 9.11 (d, 2H, ^{3}J=7.8) (6,9). ^{13}C NMR (CD_3OD-d4, δ; m.d., J/Hz): 29.28, 31.14 (4C) (13,14), 51.36 (2C) (12), 70.35 (2C) (16), 73.58 (2C) (15); 110.87 (2C) (c,e); 115.42 (1C) (d); 124.37 (4C) (3′,6,9); 125.46 (2C) (3′′); 127.58 (2C) (5, 10); 127.64 (2C) (5′); 127.85 (2C) (5′′); 130. 57 (2C) (3b,11a); 137.76 (2C) (4′); 137.84 (2C) (4′′); 144.98 (2C) (3a,11b); 148.58 (2C) (7a,7b); 149.19 (2C) (4,11); 151.41 (2C) (6′); 151.66 (2C) (6′′); 151.78 (2C) (b,f); 153.72 (1C) (2); 157.12 (2C) (2′); 157.33 (2C) (2′′). ESI-MS m/z (Iотн, (%)): 479.6 [**3**-2Cl$^-$]$^{2+}$ 100%. Calculated for $C_{49}H_{49}Cl_2N_9O_2RuS_2$ (%): C, 57.02; H, 4.79; N, 12.21; Ru, 9.79; found (%): C, 57.24; H, 4.88; N, 12.07; Ru, 9.53

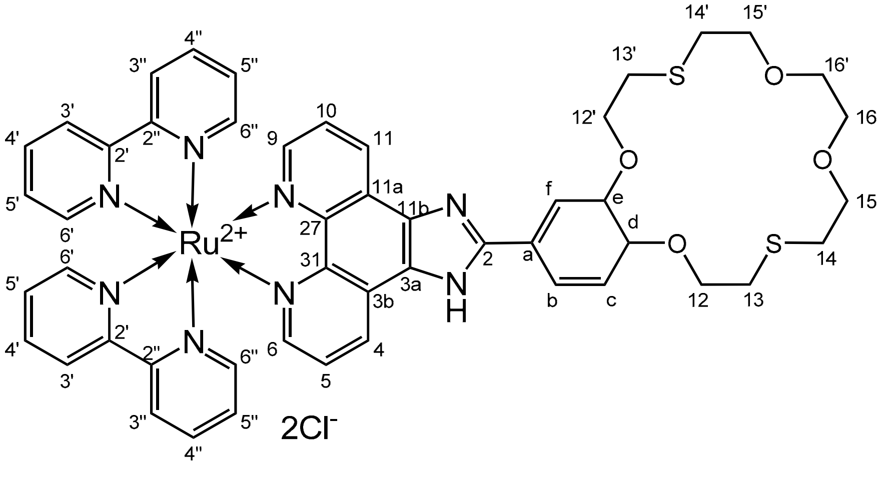

Figure S4. Structure of the *bis(2,2-bipyridine-k⁴N¹′,N¹″)[2-(2-(2,3,5,6,8,9,11,12,14,15,16a,20a dodecahydrobenzo[b] [1,4,10,13,7,16]tetraoxadithiacyclooctadecin-18-yl)-1H-imidazo[4,5- f][1,10] phenanthroline-k²N⁷,N⁸] ruthenium(II) chloride.* (**4**)

cis-bis(2,2- bipyridine)dichlororuthenium (II) hydrate (0.355 mmol, 169 mg) and ligand **2** (0.355 mmol, 200 mg) were dissolved in 10 ml absolute ethanol. The solution was placed in a tube, degassed 3 times and filled with argon. The tube was sealed and stirred at 80°C for 8 hours. Next, the tube was opened and the solvent was evaporated on a rotary evaporator. The resulting crude product was purified by flash column chromatography on alumina, eluent – a mixture of benzene:methanol in a

ratio of 4:1 to obtained 330 mg (0.315 mmol) scarlet precipitate. Yield 80 %. ^{1}H NMR (CD_3OD - d4, δ; m.d., J/Hz): 2.83 (m, 4H) H(14,14′); 2.94 (t, 2H, ^{3}J=6.2) H(13′); 3.01 (t, 2H, ^{3}J=6.5) H(13); 3.48 (s, 4H) H(16,16′); 3.60 (m, 4H) H(15,15′); 4.03 (t, 2H, ^{3}J=6.4) H(12′), 4.18 (m, 2H) H(12), 6.79 (d, 1H, ^{3}J=8.4) H(c); 7.25 (t, 2H, ^{3}J=6.7) H(5′); 7.49(t, 2H ^{3}J=6.6) H(5′′); 7.64 (d, 2H, ^{3}J=5.5) H(6′); 7.68 (dd, 2H, ^{3}J=5.11, ^{3}J=3.2) H(5,10); 7.79 (d, 1H, ^{3}J=8.2) H(b); 7.86 (d, 2H, ^{3}J=5.7) H(4,11); 7.9 (d, 2H, ^{3}J=5.2) H(6′′); 7.94 (t, 2H, ^{3}J=7.9) H(4′); 8.02 (s, 1H) H(f); 8.08 (t, 2H, ^{3}J=7.7) H(4′′); 8.62 (d, 2H, ^{3}J=8.1) H(3′); 8.67 (d, 2H, ^{3}J=8.3) H(3′′); 9.06 (d, 2H, ^{3}J=8.4) H(6,9). ^{13}C NMR (CD_3OD-d4, δ; m.d., J/Hz): 30.64 (1C) (13′); 30.69 (1C) (13); 31.21 (2C) (14,14′); 69.51 (1C) (12); 69.73 (1C) (12′); 69.86 (2C) (16,16′), 71.99 (2C) (15,15′); 112.09 (1C) (c), 119.69 (1C) (b); 124.05 (2C) (3′); 124.13 (2C) (3′′); 124.71 (2C) (5,10); 125.75 (2C) (3b, 11a); 127.32 (2C) (5′); 127.41 (2C) (5′′); 127.83 (1C) (a); 129.16 (1C) (f); 130.15 (2C) (6,9); 134,09 (2C) (3a,11b);137.46 (2C) (4′); 137.62 (2C) (4′′); 144.10 (2C) (7a,7b); 147.08 (2C) (4,11); 148.25 (1C) (d); 148.63 1(C) (e); 151.11 (2C) (6′); 151. 34 (2C) (6′′); 157,16 (2C) (2′); 157, 37 (2C) (2′′); 161.29 (1C) (2). ESI-MS m/z ($I_{отн}$, (%)): 488.0 [**4**-2Cl$^-$]$^{2+}$ 100%. Calculated for $C_{49}H_{46}Cl_2N_8O_4RuS_2$ (%): C, 56.21; H, 4.43; N, 10.70; Ru, 9.65; found (%): C, 56.39; H, 4.78; N, 10.47; Ru, 9.41

[Ag(**4**)]ClO_4. 29.7 mg of complex **4** were diluted in 24 ml of acetonitrile and 237 mkl of $AgClO_4$ acetonitrile 10^{-1} M solution was added. Yield: 17.2 mg (58%). Anal. Calcd. for $C_{49}H_{46}AgCl_3N_8O_8RuS_2$: C, 46.92; H, 3.70; Ag, 8.60; Ru, 8.06. Found: C, 49.90; H, 4.19; Ag, 6.44; Ru, 7.21. Found fractions of elements correspond to a mixture of the initial ruthenium(II) complex and a bimetallic complex in a ratio of 1:5 with a small amount of solvent inside the condensed phase. Anal. Calcd for 5·[Ag(**4**)(CH_3CN)$_5$]ClO_4+(**4**)(CH_3CN)$_5$: C, 49.73; H, 4.31; Ag, 6.31; Ru, 7.09.

[Pb(**4**)](ClO_4)$_2$. 19.6 mg of complex **4** were diluted in 20 ml of acetonitrile and 211 mkl of $Pb(ClO_4)_2$ $8.9\cdot10^{-2}$ M solution was added. Yield: 19.2 mg (69%). Anal. Calcd for $C_{49}H_{46}Cl_4N_8O_{12}PbRuS_2$: C, 40.50; H, 3.19; Pb, 14.26; Ru, 6.96; Found: C, 43.33; H, 3.66; Pb, 11.55; Ru, 6.81. Found fractions of elements correspond to a mixture of the initial ruthenium(II) complex and a bimetallic complex in a ratio of 1:5 with a small amount of solvent within the condensed phase. Anal. Calcd for 5·[Pb(**4**)(CH_3CN)$_2$](ClO_4)$_2$+(**4**)(CH_3CN)$_2$: C, 43.16; H, 3.52; Pb, 11.93; Ru, 6.98

[Cu(**3**)](ClO_4)$_2$. 23.4 mg of complex **3** were diluted in 20 ml of acetonitrile and 242 mkl of $Cu(ClO_4)_2$ $9.4\cdot10^{-2}$ M solution was added. Yield: 20.7 mg (70%). Anal. Calcd for $C_{49}H_{47}Cl_4N_9O_{10}CuRuS_2$: C, 45.52; H, 3.67; Cu, 4.92; Ru, 7.82; Found: C, 47.53; H, 3.99; Cu, 3.95; Ru, 7.21. Found fractions of elements correspond to a mixture of the initial ruthenium(II) complex and a bimetallic complex in a ratio of 1:7 with a small amount of solvent within the condensed phase. Anal. Calcd for 7·[Cu(**3**)(CH_3CN)$_3$](ClO_4)$_2$+(**4**)(CH_3CN)$_3$: C, 47.77; H, 4.08; Cu, 4.02; Ru, 7.31
